# The intersectional impact of sex and social factors on subjective health: analysis of the Canadian longitudinal study on aging (CLSA)

**DOI:** 10.1186/s12877-021-02412-6

**Published:** 2021-08-28

**Authors:** Afshin Vafaei, Janelle Yu, Susan P. Phillips

**Affiliations:** 1 Department of Family Medicine, Queen’s University, Kingston, ON Canada; 2 Department of Public Health Sciences, Queen’s University, Centre for Studies in Primary Care, 220 Bagot St, Kingston, ON K7L 5E9 Canada

**Keywords:** Sex differences, Self-rated health, CLSA, Intersectionality, Social determinants

## Abstract

**Background:**

Self-rated health (SRH) is a widely validated measure of the general health of older adults. Our aim was to understand what factors shape individual perceptions of health and, in particular, whether those perceptions vary for men and women and across social locations.

**Methods:**

We used data from the Canadian Longitudinal Study on Aging (CLSA) of community-dwelling adults aged 45 to 85. SRH was measured via a standard single question. Multiple Poisson regression identified individual, behavioural, and social factors related to SRH. Intersections between sex, education, wealth, and rural/urban status, and individual and joint cluster effects on SRH were quantified using multilevel models.

**Results:**

After adjustment for relevant confounders, women were 43% less likely to report poor SRH. The strongest cluster effect was for groupings by wealth (21%). When wealth clusters were subdivided by sex or education the overall effect on SRH reduced to 15%. The largest variation in SRH (13.6%) was observed for intersections of sex, wealth, and rural/urban status. In contrast, interactions between sex and social factors were not significant, demonstrating that the complex interplay of sex and social location was only revealed when intersectional methods were employed.

**Conclusions:**

Sex and social factors affected older adults’ perceptions of health in complex ways that only became apparent when multilevel analyses were carried out. Utilizing intersectionality analysis is a novel and nuanced approach for disentangling explanations for subjective health outcomes.

**Supplementary Information:**

The online version contains supplementary material available at 10.1186/s12877-021-02412-6.

## Background

### Conceptualization of self-rated health

The overarching question we examine in this study is what individual and contextual characteristics shape the subjective rating of health among older adults. Self-rated health (SRH) is a widely used predictor of mortality and physical functioning in general [1] and, particularly, among older populations [2]. The usual measure is a single question asking for a rating of one’s health using a five-point scale ranging from poor to excellent. This is often dichotomized into ‘good’ and ‘poor’. The simplicity of this measure, its demonstrated validity [3], and its significant linear association with objective health indicators such as physical functioning [4] explain SRH’s wide acceptance [5]. Some authors argue, however, that the subjective aspect of SRH is increasingly problematic when older populations are studied [6]. With aging, individual expectations and standards of good health evolve. Both perceptions of normal health status for a particular age and awareness of diagnoses that lack symptoms but raise the spectre of illness (eg hypertension) play important roles as reference points for an individual’s self-rating. Older adults also may rate their health relative to their age cohort and related expectation rather than to some absolute standard [7]. This shift in comparative baseline may be a way of coping with and adapting to declining health, but also makes tracking of SRH across the life-course and its reliability as an indicator of older adults’ objective health challenging. Still others have contested these presumed, age-related measurement modifications [7].

In addition to shifts arising from individual psychology, and expectations and perceptions of health over the life-course, older adults’ SRH may also be shaped by norms and values aligned with group affiliation, whether those groups are social, cultural, or based on innate traits like sex. For example, when they rate their health the components considered by older men and women seem to be different [8]. Men tend to focus specifically on physical well-being in making their determination whereas women take a broader view, considering mental health and levels of physical activity and function as contributing factors [8]. According to a comprehensive framework proposed by Jylhä [6], what constitutes ‘health’ can also vary with geography and culture. At a contextual level, cultural norms and social roles affect self-assessments of health. For example, after controlling for related sociodemographic and health characteristics, Italians, Dutch men, and non-Hispanic whites assessed their health more positively than Finns, Lithuanian men, and Hispanics, respectively [6], while Germans underrated their health when compared to Danes or Swedes [9]. Other authors suggest that Americans are ‘health optimistic’, finding that when compared to their Japanese counterparts and despite presenting poorer measured health outcomes, Americans rated their health more highly [10].

### Sex and SRH across different social circumstances

Among older adults, women generally report lower SRH than do men, however this finding varies across countries [11–15]. Even within countries findings are inconsistent; no sex/gender differences were found in some sample populations from Canada and Colombia [16, 17]. It may be that culturally-based gender norms influence how women and men weigh components of SRH. Although this has not been studied, in theory, men in more traditional environments might consider physical robustness as central to better SRH while dismissing mental health as a contributing factor. In Canada, relatively expansive and egalitarian gender roles and older women’s educational and socio-economic attainment that often meets that of age-matched men, may shift perceptions, particularly among women, of SRH [18]. We hypothesize that conventions, definitions, and references used to assess health will vary across sex but also with intersections of sex and other social locations. This represents a gender effect rather than only an effect of sex.

Characteristics that fit within a social determinants of health framework such as education [13], income [19], race [20], rural/urban place of residence [21], marital status [22], and life-course adversities [23] all underpin perceptions of health among older adults [24].

The relative effect of inequalities in social circumstances on subjective health tends to decline with age, while the impact of contextual factors such as culture and geography becomes more prominent, a phenomenon referred to as ‘age-as-leveler’ [25]. As a result, subjective health status differences may narrow among older people occupying similar social locations [26].

### Intersectionality: sex, social factors, a measure of ‘gender’

To some extent, studies that perform sex-stratified analyses are able to differentiate effects of sex and social factors. For example, SRH effects of either income [27] or rurality [28] are sometimes stronger in women, whereas the effect of marital status [29], deprivation [30], childhood and lifetime cumulative socio-economic status (SES) [31] and education [32] appear to be stronger among men. However, concomitant social factors such as race and education work with sex in interconnected and complex ways to affect health outcomes. These are often not simply the additive or multiplicative effects of interactions. Adopting an intersectionality framework offers a more nuanced understanding of how complex, co-existing effects of sex and social locations determine subjective health by quantifying ‘between social location variations’ and ‘within social location heterogeneities’. For example, in a study of intertwined effects of five dimensions of social location (sex, race, income, education, and age) on body mass index among U.S. adults, the intersectional approach of multilevel analysis provided advantages over conventional models by identifying heterogeneities of risk attributable to within social location variations [33]. Such a framework should aid in addressing social opportunities and constraints arising from sex, that is, in addressing gender, a much theorized but difficult to measure social determinant of health [34, 35]. With roots in sociology and the study of inequality, intersectionality frameworks assume that interlocking and overlapping characteristics such as race, income, education or sex/gender jointly alter subjective and objective health outcomes. The focus of our study is sex and the three social locations of education, wealth, and rural/urban residence, all widely reported independent predictors of SRH [31], and whether these four predictors intersect in shaping SRH among Canadian adults age 45 + .

Although consensus is yet to be reached, various quantitative techniques for studying intersections of, for example, sex and social factors have recently been demonstrated. Utilizing structural equation modelling (SEM), Wang et al. [36] found that SES not only directly influences the subjective health status of men and women differently, but also has differential indirect effects across sex groups through interactions with other social circumstances. Using European data Arpino et al. [37] examined the mediating effect of educational attainment on how early-life conditions shape older adults’ SRH and found a stronger effect among men [37]. Using decomposition analysis techniques that partitioned gender inequities in SRH by SES, measures such as education and employment in Europe [38] and India [39] it appeared that the social vulnerability of older women in terms of educational attainment or access to well-paid jobs contributed to their poorer SRH. Multilevel (ML) analysis techniques offer another option for quantitative examination of intersectionality and have been used in several studies [40–42]. ML models typically account for the ‘nesting’ or ‘clustering’ of individuals within geographic settings such as neighbourhoods. However, clusters examined can also include groupings by sex or social strata defined by levels of wealth and/or education attainment. Those sharing a cluster may well share certain characteristics that shape values and behaviors. This commonality violates the assumption that each participant in a study is independent of all others, an assumption that is central to ordinary regression analysis. Multilevel analyses assess combined effects of, for example, sex and social factors simultaneously and interactively, not simply as additive or multiplicative interactions [42] and therefore are able to identify independent dimensions of stratification by social factors. ML analyses quantify cluster effects by estimating indicators such as the intra-class correlation coefficient (ICC); defined as the ratio of the between-cluster variance to the total variance. A large ICC suggests that variation between clusters has an important impact on an outcome and should be taken into account in etiological analyses. The Median Odds Ratio (MOR) is another measure of clustering. MOR quantifies between-cluster variations by exhaustively comparing any two randomly chosen persons, one from each cluster, offering more interpretable information for discrete outcomes in form of an Odds Ratio [43, 44]. A larger MOR indicates higher variability between clusters.

Research on how intersections of sex and social factors shape perceived health of older adults is scarce. To address this gap our objectives were to 1) estimate the unbiased impact of sex and social circumstances on reported SRH in Canadian men and women age 45+; 2) explore interactions of three key dimensions of social identity (education, wealth, and rural/urban status) on the sex-SRH relationship; 3) explore intersections of sex and social factors, that is, of gender and SRH. By comparing findings across these analytic designs we hope to form a nuanced picture of how an older adult’s multiple individual and social facets intersect to shape that health.

## Methods

### Settings and participants

The Canadian Longitudinal study on Aging (CLSA) includes a random sample of 30,097 community-dwelling adults aged 45 to 85 residing within a 25- to 50-km radius of 1 of the 11 data collection sites in 7 Canadian provinces (Raina et al., 2019). Individuals living in an institution or on a First Nations reserve or settlement, who were full-time members of the Canadian Armed Forces, were unable to speak French or English, or had cognitive impairment that could hamper answering of basic personal questions were excluded.

### Sampling strategy

For the Comprehensive Cohort of the CLSA two sampling strategies were used. To recruit from provincial health registries (14% of the sample), randomly chosen eligible persons were sent a consent form to sign and return. For those recruited through random digit dialing (86% of the sample), a random sample of landline telephone numbers was selected for a given geographic area. After establishing eligibility among those answering calls, informed consent was obtained. The CLSA sample was stratified within provinces according to age group, sex, and distance from the data collection site, to ensure adequate representation of various demographic groups.

### Data collection

After extensive training to ensure standardized data collection, interviewers administered questionnaires at participants’ homes or at a data collection site. Physical examinations were conducted at the data collection site. Data were collected between 2010 and 2015.

### Assessment of self-rated health (SRH)

Participants were asked to assess their health by answering a standard 1-item self-report question, ‘Would you say your health is excellent, very good, good, fair, poor?’ We collapsed the first three categories into a ‘good’ and the last two into a ‘poor’ SRH category and included SRH and a dichotomous outcome variable in the analysis.

### Assessment of sex and main social factors (dimensions of social identity)

Information on sex, highest educational attainment (less than secondary school, secondary school graduation, some post-secondary, post-secondary graduation) and wealth (whether income fulfills basic needs) was collected via direct questions. Participants’ residence postal codes were linked to dissemination area to classify place of residence into ‘urban’ and ‘rural’. In Canada, the best proxy measure for place of residence in terms of studying health outcomes is rural/urban status [45].

### Covariates

We wanted to identify all possible available predictors for perceptions of health in old age, therefore, we included a large number of variables in initial descriptive analyses.

*Socio-demographic characteristics* included age in years, marital status (currently married or common law, windowed, divorced, separated, single), country of birth, province, and household income as a categorical variable (<$20,000, $20,000–$49,999, $50,000–$99,999, $100,000–$149,999, ≥$150,000, Don’t know/No answer/Refused). We also included ‘frequency of community-related activity participation’ as the measure of social participation and results of the medical outcomes study (MOS) social support scale [46] to indicate social support (see Appendix A for details).

*Lifestyle factors* included body mass index (BMI) classification [underweight< 18.5 kg/m2, normal weight (18.5–25 kg/m2), overweight (25–30 kg/m2), obese (> 30 kg/m2)], smoking history (< 100 cigarettes in lifetime or never smoked, former smoker, current smoker), drinking behavior in the past 12 months [did not drink in the last 12 months, occasional drinker, regular drinker (at least once a month)] and frequency of alcohol consumption (number of drinks per week). Nutritional risk was measured using the AB SCREENTM II (Abbreviated Seniors in the Community Risk Evaluation for Eating and Nutrition II) scale [47] that included questions on weight change, eating habits, difficulty eating, fruit, vegetable and fluid consumption, meal satisfaction, frequency of fast-food consumption, coffee and tea consumption, and food security. A modified version of the Physical Activity Scale for the Elderly (PASE) tool [48] was used to measure frequency of physical activity in the week prior to the interview and the amount of physical activity associated with one’s work or volunteer activities.

*Health status* variables included questions about vision, hearing, as well as a modification of the Activities of Daily Living (ADL) and Instrumental Activities of Daily Living (IADL) questions of the OARS Multidimensional Assessment Questionnaire [49]. Additionally, the 10-item version of the Centre for Epidemiologic Studies Depression (CESD) scale was used to measure depression [50]. Hand grip strength was measured objectively using a hand dynamometer (Appendix A). Finally, we included data on receiving and giving informal and formal care in the analysis.

### Statistical analysis

Characteristics of participants were described across sex and SRH groups by calculation of means and standard deviations of continuous variables and frequency distributions of categorical variables. The significance of bivariate associations between covariates and sex and the outcome (SRH) were evaluated using t-test and Chi-square tests where appropriate. To estimate the unbiased effect of sex on the probabilities of reporting good SRH, we used Poisson regression models with robust variance. The most parsimonious models were constructed following the change in estimate method [51] to adjust only for true confounders. All variables that were significantly related to SRH or sex were entered in an initial *main effect* model. We started to trim this model by removing the variables with largest *p* values one by one. If the removal of a variable caused more than a 10% change in the sex-SRH association, the variable would be entered back into the model, even if the related *p* value were larger than 0.05. Results were reported as prevalence rate ratios (PRR) and corresponding 95% confidence intervals (95% CI) which are proper epidemiological effect estimates for cross-sectional data. We also tested for interactions between sex and other selected social factors (education, wealth, rural/urban status) for the outcome of SRH. None was significant; nevertheless, results were reported for the whole sample as well as stratified by sex. Following precedents [40, 41] to test the intersecting effects of social factors and sex on SRH we constructed logistic multi-level models to calculate Intra-class Correlation Coefficients (ICC) and Median Odds Ratios (MOR) for each of the four selected social identity factors and their combinations. We did not include the main effects of social strata in the model and only constructed a set of empty models (intercept only) in which the random effect clusters were defined by the four social identity strata (sex, education, wealth, rural/urban status).

All analyses were performed using SAS version 9.4 (SAS Institute, Cary, NC, USA).

## Results

*Descriptive:* Almost half of all participants were female and, overall, more than 90% perceived their health as good. There was no statistically significant difference in this perception between men and women (*p* = 0.066). With the exception of availability of social support, province of residence and nutrition, distributions of all other variables differed significantly for men and women (Table 1).

**Table 1:** Characteristics of the CLSA sample by sex

Variable	Description	Female	Male	P.value
Sex		15,320 (50.90%)	14,777 (49.10%)	–
Age	In years (mean, SD)	62.8 (10.2)	63.2 (10.3)	0.001
Self-rated Health	Good health	13,939 (91.05%)	13,353 (90.44%)	0.066
	Poor health	1370 (8.95%)	1412 (9.56%)	
Self-rated Mental health	Good health	14,427 (94.24%)	13,990 (94.77%)	0.046
	Poor health	881 (5.76%)	772 (5.23%)	
Chronic conditions	At least 1 chronic condition	14,347 (94.17%)	13,286 (90.68%)	< 0.0001
	No chronic conditions	888 (5.83%)	1365 (9.32%)	
Social support availability	Higher = better (mean, SD)	99.4 (130.1)	99.8 (130.4)	0.125
Social support participation	Did not participate in community related activity	16 (0.10%)	20 (0.14%)	< 0.0001
	Participated in community related activity at least 1/year	173 (1.13%)	254 (1.72%)	
	Participated in community related activity at least 1/month	1605 (10.48%)	2172 (14.70%)	
	Participated in community related activity at least 1/week	10,623 (69.34%)	9904 (67.02%)	
	Participated in community related activity at least 1/day	2876 (18.77%)	2397 (16.22%)	
Country of birth	Canada	12,774 (83.40%)	11,870 (80.33%)	< 0.0001
	Other	2543 (16.60%)	2907 (19.67%)	
Province of residence	Alberta	1517 (9.90%)	1440 (9.74%)	0.294
	British Columbia	3158 (20.61%)	3096 (20.95%)	
	Manitoba	1594 (10.40%)	1519 (10.28%)	
	Newfoundland/Labrador	1132 (7.39%)	1082 (7.32%)	
	Nova Scotia	1549 (10.11%)	1529 (10.35%)	
	Ontario	3207 (20.93%)	3211 (21.73%)	
	Quebec	3163 (20.65%)	2900 (19.63%)	
Location	Urban	13,828 (91.48%)	13,467 (92.22%)	0.032
	Rural	1288 (8.52%)	1136 (7.78%)	
Marital status	Single	1456 (9.50%)	1198 (8.11%)	< 0.0001
	Married/in a common law relationship	9160 (59.59%)	11,491 (77.76%)	
	Widowed	2079 (13.57%)	730 (4.94%)	
	Divorced	2180 (14.23%)	1005 (6.80%)	
	Separated	440 (2.87%)	350 (2.37%)	
Education level	Less than secondary school graduation	918 (5.99%)	725 (4.91%)	< 0.0001
	Secondary school graduation, no post-secondary	1610 (10.51%)	1229 (8.32%)	
	Some post-secondary education	1164 (7.60%)	1074 (7.27%)	
	Post- secondary degree/diploma	11,609 (75.78%)	11,718 (79.30%)	
Wealth (how well does your income satisfy your basic needs?)	Very well	7680 (50.13%)	7848 (53.11%)	< 0.0001
	Adequately	5387 (35.16%)	5090 (34.45%)	
	With some difficulty	1042 (6.80%)	781 (5.29%)	
	Not very well	250 (1.63%)	220 (1.49%)	
	Totally inadequately	117 (0.76%)	83 (0.56%)	
Income	Less than $20,000	3174 (20.72%)	1199 (8.11%)	< 0.0001
	$20,000 or more, but less than $50,000	6053 (39.51%)	4485 (30.35%)	
	$50,000 or more, but less than $100,000	4062 (26.51%)	5634 (38.13%)	
	$100,000 or more, but less than $150,000	738 (4.82%)	1779 (12.04%)	
	$150,000 or more	326 (2.13%)	1139 (7.71%)	
Smoking	Daily smoker	1261 (8.23%)	1449 (9.81%)	< 0.0001
	Occasional smoker or former daily smoker	7783 (50.80%)	6459 (43.71%)	
	< 100 cigarettes in lifetime/non smoke	6276 (40.97%)	6868 (46.48%)	
Alcohol consumption	Regular drinker (at least once/month)	10,567 (68.98%)	11,672 (78.99%)	< 0.0001
	Occasional drinker	2409 (15.72%)	1296 (8.77%)	
	Did not drink in the last 12 months	1884 (12.30%)	1543 (10.44%)	
Alcohol consumption frequency	Almost every day	1904 (12.81%)	2941 (20.27%)	< 0.0001
	4–5 times/week	1310 (8.82%)	1684 (11.60%)	
	2–3 times/week	2847 (19.16%)	3286 (22.65%)	
	1 time/week	1684 (11.33%)	1612 (11.11%)	
	Occasionally	5231 (35.20%)	3444 (23.73%)	
	Never	1884 (12.68%)	1543 (10.63%)	
Nutrition	Higher = better (mean, SD)	37.3 (18.5)	37.5 (19.5)	0.191
Physical activity- frequency last week	Never	10,492 (68.49%)	9848 (66.64%)	< 0.0001
	Seldom (1–2 days)	1649 (10.76%)	1322 (8.95%)	
	Sometimes (3–4 days)	1422 (9.28%)	1610 (10.90%)	
	Often (5–7 days)	1075 (7.02%)	1352 (9.15%)	
Physical activity- hours/day	< 30 min	1905 (12.43%)	1957 (13.24%)	< 0.0001
	30 min to 1 h	1391 (9.08%)	1376 (9.31%)	
	1 h to 2 h	763 (4.98%)	844 (5.71%)	
	2 h to 4 h	69 (0.45%)	85 (0.58%)	
	4 h +	9 (0.06%)	14 (0.09%)	
Physical activity at work	Mainly sitting with slight arm movements	2882 (18.81%)	3238 (21.91%)	< 0.0001
	Sitting and standing with some walking	3090 (20.17%)	2652 (17.95%)	
	Walking with some light handling of materials	1888 (12.32%)	1830 (12.38%)	
	Walking and heavy manual work	131 (0.86%)	462 (3.13%)	
ADL+ IADL	Higher = independent for more ADL and IADL (mean, SD)	0.0082 (0.1710)	0.0043 (0.0702)	0.002
Depression	Higher = more depression symptoms (mean, SD)	3.9 (2.6)	3.5 (2.6)	< 0.0001
BMI	Underweight	164 (1.07%)	53 (0.36%)	< 0.0001
	Normal	5331 (34.80%)	3532 (23.90%)	
	Overweight	5302 (34.61%)	6786 (45.92%)	
	Obese Class I	2663 (17.38%)	3157 (21.36%)	
	Obese Class II	1131 (7.38%)	847 (5.73%)	
	Obese Class III	659 (4.30%)	336 (2.27%)	
Vision	Excellent	3362 (21.95%)	3488 (23.60%)	
	Very Good	5975 (39.00%)	5775 (39.08%)	0.001
	Good	4758 (31.06%)	4414(29.87%)	
	Fair	1010 (6.59%)	947 (6.41%)	
	Poor	200 (1.31%)	149 (1.01%)	
Hearing	Excellent	3931 (25.665)	2735 (18.51%)	< 0.0001
	Very Good	5404 (35.27%)	4686 (31.71%)	
	Good	4700 (30.68%)	5179 (35.05%)	
	Fair	1107 (7.22%)	1863 (12.61%)	
	Poor	164 (1.07%)	301 (2.04%)	
Grip strength	Higher = stronger grip (mean, SD)	26.5130 (6.0016)	43.7429 (9.7894)	< 0.0001
Formal health care services	Received formal health care services	902 (5.89%)	560 (3.79%)	< 0.0001
	Did not receive formal health care services	14,411 (94.07%)	14,207 (96.14%)	
Informal health care services	Received informal health care services	2043 (13.34%)	1310(8.87%)	
	Did not receive informal health care services	13,271 (86.63%)	13,460 (91.09%)	
Care services	Did not receive any health care services	12,857 (83.92%)	13,170 (89.12%)	< 0.0001
	Received formal health care services only	409 (2.67%)	282 (1.91%)	
	Received informal health care services only	1551 (10.12%)	1033 (6.99%)	
	Received both formal and informal health care services	491 (3.20%)	277 (1.87%)	
Care giving	Did provide assistance for others	7215 (47.10%)	5828 (39.44%)	< 0.0001
	Did not provide assistance for others	8093 (52.83%)	8939 (60.49%)	
Hours care giving	Average hours/week caregiving for others (mean, SD)	15.9 (28.1)	11.3 (21.7)	

1. Numbers inside parentheses are column percentage or SD

2. *P* values are from t-test or chi-square test where appropriate.

Frequencies of reporting good SRH also varied significantly across all other characteristics considered with the exception of physical activity at work, country of birth, province of residence and rural/urban status (Table 2).

**Table 2:** Characteristics of the CLSA sample by the SRH Status

Variable	Description	Good SRH	Poor SRH	*P*.value
Sex	Female	13,939 (91.05%)	1370 (8.95%)	0.0661
	Male	13,353 (90.44%)	1412 (9.56%)	
Age	In years (mean, SD)	62.9 (10.2)	63.4 (10.4)	0.0004
Self-rated Mental health	Good health	26,448 (93.13%)	1951 (6.87%)	0.0107
	Poor health	830 (50.27%)	821 (49.73%)	
Chronic conditions	At least 1 chronic condition	24,878 (90.10%)	2733 (9.90%)	< 0.0001
	No chronic conditions	2216 (98.40)	36 (1.60%)	
Social support availability	Higher = better (mean, SD)	99.1 (125.4)	104.5 (169.7)	< 0.0001
Social support participation	Did not participate in community related activity	31 (68.89%)	14 (31.11%)	
	Participated in community related activity at least 1/year	306 (71.66%)	121 (28.34%)	< 0.0001
	Participated in community related activity at least 1/month	3205 (84.97%)	567 (15.03%)	< 0.0001
	Participated in community related activity at least 1/week	18,808 (91.69%)	1704 (8.31%)	
	Participated in community related activity at least 1/day	4906 (93.08%)	365 (6.92%)	
Country of birth	Other	3086 (91.33%)	293 (8.67%)	0.4885
	Canada	24,203 (90.68%)	2489 (9.32%)	
Province of residence	Alberta	2680 (90.72%)	274 (9.28%)	0.1852
	British Columbia	5647 (90.35%)	603 (9.65%)	
	Manitoba	2825 (90.81%)	286 (9.19%)	
	Newfoundland/Labrador	1999 (90.33%)	214 (9.67%)	
	Nova Scotia	2783 (90.53%)	291 (9.47%)	
	Ontario	5878 (91.66%)	535 (8.34%)	
	Quebec	5480 (90.44%)	579 (9.56%)	
Location	Urban	24,732 (90.68%)	2542 (9.32%)	0.0799
	Rural	2224 (91.82%)	198 (8.18%)	
Marital status	Single	2305 (86.85%)	349 (13.15%)	< 0.0001
	Married/in a common law relationship	19,004 (92.09%)	1632 (7.91%)	
	Widowed	2520 (89.84%)	285 (10.16%)	
	Divorced	2763 (86.86%)	418 (13.14%)	
	Separated	692 (87.59%)	98 (12.41%)	
Education level	Less than secondary school graduation	1317 (80.30%)	323 (19.70%)	< 0.0001
	Secondary school graduation, no post-secondary	2536 (89.36%)	302 (10.64%)	
	Some post-secondary education	1959 (87.61%)	277 (12.39%)	
	Post- secondary degree/diploma	21,440 (91.98%)	1870 (8.02%)	
Wealth (how well does your income satisfy your basic needs?)	Very well	14,676 (94.57%)	843 (5.43%)	< 0.0001
	Adequately	9388 (89.69%)	1079 (10.31%)	
	With some difficulty	1478 (81.08%)	345 (18.92%)	
	Not very well	328 (69.78%)	142 (30.21%)	
	Totally inadequately	119 (59.50%)	81 (40.50%)	
Income	Less than $20,000	3522 (80.56%)	850 (19.44%)	< 0.0001
	$20,000 or more, but less than $50,000	9529 (90.52%)	998 (9.48%)	
	$50,000 or more, but less than $100,000	9102 (93.93%)	588 (6.07%)	
	$100,000 or more, but less than $150,000	2400 (95.35%)	117 (4.65%)	
	$150,000 or more	1395 (95.29%)	69 (4.71%)	
Smoking	Daily smoker	2243 (82.80%)	466 (17.20%)	< 0.0001
	Occasional smoker or former daily smoker	13,230 (92.96%)	1002 (7.04%)	
	< 100 cigarettes in lifetime/non-smoker	11,818 (89.99%)	1314 (10.01%)	
Alcohol consumption	Regular drinker (at least once/month)	20,699 (93.12%)	1529 (6.88%)	< 0.0001
	Occasional drinker	3134 (84.75%)	564 (15.25%)	
	Did not drink in the last 12 months	2828 (82.62%)	595 (17.38%)	
Alcohol consumption frequency	Almost every day	4518 (93.41%)	319 (6.59%)	< 0.0001
	4–5 times/week	2838 (94.79%)	156 (5.21%)	
	2–3 times/week	5793 (94.49%)	338 (5.51%)	
	1 time/week	3051 (92.57%)	1035 (7.43%)	
	Occasionally	7633 (88.06%)	1035 (11.94%)	
	Never	2828 (82.62%)	595 (17.38%)	
Nutrition	Higher = better	37.97 (18.21)	30.97 (24.73)	< 0.0001
Physical activity- frequency last week	Never	18,305 (90.06%)	2021(9.94%)	< 0.0001
	Seldom (1–2 days)	2791 (93.97%)	179 (6.03%)	
	Sometimes (3–4 days)	2887 (95.28%)	143 (4.72%)	
	Often (5–7 days)	2236 (92.24%)	188 (7.76%)	
Physical activity- hours/day	< 30 min	3584 (92.90%)	274 (7.10%)	0.00004
	30 min to 1 h	2610 (94.36%)	156 (5.64%)	
	1 h to 2 h	1537 (95.70%)	69 (4.30%)	
	2 h to 4 h	148 (96.10%)	6 (3.90%)	
	4 h +	20 (86.96%)	3 (13.04%)	
Physical activity at work	Mainly sitting with slight arm movements	5730 (93.67%)	387 (6.33%)	0.1775
	Sitting and standing with some walking	5410 (94.22%)	332 (5.78%)	
	Walking with some light handling of materials	3470 (93.38%)	246 (6.62%)	
	Walking and heavy manual work	547 (92.40%)	45 (7.60%)	
ADL+ IADL	Higher = independent for more ADL and IADL (mean, SD)	0.0051 (0.1304)	0.0176 (0.1421)	< 0.0001
Depression	Higher = more depression symptoms (mean, SD)	3.6 (2.6)	5.1 (2.6)	< 0.0001
BMI	Underweight	177 (81.57%)	40 (18.43%)	< 0.0001
	Normal	8352 (94.32%)	503 (5.68%)	
	Overweight	11,260 (93.23%)	818 (6.77%)	
	Obese Class I	5110 (87.86%)	706 (12.14%)	
	Obese Class II	1610 (81.40%)	368 (18.60%)	
	Obese Class III	692 (69.92%)	302 (30.38%)	
Vision	Excellent	6435 (94.04%)	408 (5.96%)	< 0.0001
	Very Good	10,963 (93.37%)	778 (6.63%)	
	Good	8102 (88.36%)	1067 (11.64%)	
	Fair	1521(77.72%)	436 (22.28%)	
	Poor	254 (73.41%)	92 (26.59%)	
Hearing	Excellent	6189 (92.91%)	472 (7.09%)	< 0.0001
	Very Good	9412 (93.30%)	676 (6.70%)	
	Good	8842 (89.62%)	1024 (10.38%)	
	Fair	2472 (83.34%)	494 (16.66%)	
	Poor	356 (76.56%)	109 (23.44%)	
Grip strength	Higher = stronger grip (mean, SD)	35.4 (11.8)	33.1 (11.8)	< 0.0001
Formal health care services	Received formal health care services	1022 (70.00%)	438 (30.00%)	< 0.0001
	Did not receive formal health care services	26,256 (91.81%)	2342 (8.19%)	
Informal health care services	Received informal health care services	2533 (75.66%)	815 (24.34%)	< 0.0001
	Did not receive informal health care services	24,751 (92.65%)	1963 (7.35%)	
Care services	Did not receive any health care services	24,214 (93.09%)	1797 (6.91%)	< 0.0001
	Received formal health care services only	526 (76.23%)	164 (23.77%)	
	Received informal health care services only	2037 (78.95%)	543 (21.05%)	
	Received both formal and informal health care	495 (64.54%)	272 (35.46%)	
Care giving	Did provide assistance for others	11,907 (91.34%)	1129 (8.66%)	0.0019
	Did not provide assistance for others	15,364 (90.29%)	1652 (9.71%)	
Hours care giving	Average hours/week caregiving for others (mean, SD)	13.6 (25.1)	16.4 (30.3)	< 0.0001

1. Numbers inside parentheses are row percentage or SD

2. *P* values are from t-test or chi-square test where appropriate

The unadjusted Poisson model showed that compared to men, women were 7% less likely to report poor SRH (PRR = 0.93; 95% CI: 0.87, 1.00). After adjustment for true confounders, identified via a well-formulated model specified following change in estimate processes, the bivariate marginally significant association between sex and SRH became significant and much stronger (PRR = 0.57; 95%CI: 0.51, 0.64). In other words, after accounting for true confounders women were 43% less likely to report poor SRH. When findings for women and men were separated in sex stratified models (Table 3) the following characteristics or variables aligned significantly with reports of poor health for both men and women: more chronic conditions, lower social participation, lower wealth (income adequacy), poorer nutrition, depression, impaired hearing, and weaker grip.

**Table 3:** Results of sex-stratified Poisson models (outcome: poor SRH)

Variable	Description	PRR All	PRR (Men)	PRR (Women)
Sex	Female vs male	0.57 (0.51–0.64)		
Age	In years	0.983 (0.978–0.987)	0.980 (0.973–0.988)	0.982 (0.975–0.989)
Chronic conditions	At least 1 chronic condition	5.95 (2.73–5.70)	3.15 (1.96–5.07)	5.30 (2.44–11.52)
	No chronic conditions	Ref.	Ref.	Ref.
Social support availability	Availability of social support	0.994 (0.992–0.996)	0.994 (0.990–0.998)	0.992 (0.989–0.996)
Social support participation	Did not participate in community related activity	1.50 (0.79–2.79)	1.79 (0.57–5.63)	1.36 (0.62–2.69)
	Participated in community related activity at least 1/year	1.54 (1.20–2.79)	1.51 (1.05–2.18)	2.00 (1.35–2.99)
	Participated in community related activity at least 1/month	1.58 (1.37–1.82)	1.52 (1.21–1.91)	1.72 (1.36–2.19)
	Participated in community related activity at least 1/week	1.21 (1.07–1.38)	1.09 (0.89–1.32)	1.34 (1.09–1.64)
	Participated in community related activity at least 1/day	Ref.	Ref.	Ref.
Education level	Less than secondary school graduation	1.30 (1.13–1.49)	–	1.35 (1.10–1.65)
	Secondary school graduation, no post-secondary	0.98 (0.86–1.11)	–	1.05 (0.86–1.28)
	Some post-secondary education	1.10 (0.97–1.26)	–	1.19 (0.97–1.46)
	Post- secondary degree/diploma	Ref.	–	Ref.
Wealth (how well does your income satisfy your basic needs?)	Totally inadequately	1.76 (1.40–2.23)	1.68 (1.13–2.51)	1.69 (1.18–2.41)
	Not very well	1.77 (1.47–2.13)	1.81 (1.34–2.45)	1.56 (1.14–2.14)
	With some difficulty	1.34 (1.05–1.54)	1.18 (0.93–1.50)	1.35 (1.09–1.67)
	Adequately	1.24 (1.13–1.36)	1.12 (0.96–1.30)	1.26 (1.08–1.48)
	Very well	Ref.	Ref.	Ref.
Income	Less than $20,000	1.32 (1.12–1.56)	1.75 (1.30–2.34)	1.24 (0.96–1.59)
	$20,000 or more, but less than $50,000	0.97 (0.83–1.13)	1.33 (1.03–1.72)	0.86 (0.67–1.11)
	$50,000 or more, but less than $100,000	0.85 (0.72–0.99)	1.02 (0.79–1.32)	0.77 (0.56–0.97)
	$100,000 or more, but less than $150,000	0.74 (0.59–0.93)	0.93 (0.68–1.28)	0.61 (0.36–1.03)
	$150,000 or more	Ref.	Ref.	Ref.
Smoking	Daily smoker	1.41 (1.24–1.60)	1.41 (1.15–1.73)	1.50 (1.24–1.83)
	Occasional smoker or former daily smoker	1.25 (1.15–1.34)	1.32 (1.14–1.52)	1.05 (1.02–1.34)
	< 100 cigarettes in lifetime/non-smoker	Ref.	Ref.	Ref.
Alcohol consumption	Regular drinker (at least once/month)	0.65 (0.59–0.72)	0.78 (0.66–0.93)	0.59 (0.50–0.69)
	Occasional drinker	0.91 (0.81–1.02)	1.07 (0.86–1.34)	0.84 (0.71–0.99)
	Did not drink in the last 12 months	Ref.	Ref.	Ref.
Nutrition	Higher = healthier nutrition	0.997 (0.995–0.998)	0.996 (0.993–0.998)	0.996 (0.994–0.998)
Depression	Measure of depressive symptoms	1.019 (1.014–1.024)	1.023 (1.022–1.036)	1.021 (1.015–1.027)
BMI	Underweight	1.77 (1.25–2.51)	2.10 (1.20–3.66)	1.69 (1.01–2.82)
	Normal	Ref.	Ref.	Ref.
	Overweight	1.11 (0.98–1.24)	1.00 (0.83–1.21)	1.31 (1.08–1.58)
	Obese	2.06 (1.85–2.29)	2.22 (1.86–2.65)	2.28 (1.91–2.72)
Vision	Poor	1.76 (1.56–1.98)	–	1.80 (1.48–2.18)
	Good	1.38 (1.26–1.51)	–	1.33 (1.15–1.54)
	Excellent	Ref.	–	Ref.
Hearing	Poor	1.78 (1.59–1.98)	1.99 (1.69–2.33)	1.76 (1.45–2.12)
	Good	1.22 (1.11–1.34)	1.39 (1.20–1.62)	1.16 (1.00–1.33)
	Excellent	Ref.	Ref.	Ref.
Grip strength	In Kg	0.983 (0.977–0.988)	0.986 (0.979–0.993)	0.975 (0.963–0.987)
Care services	Received both formal and informal health care services	2.87 (2.51–3.29)	3.27 (2.55–4.18)	2.71 (2.20–3.32)

*PRR* prevalence rate ratio

Numbers inside parentheses are 95%CI

Somewhat unexpectedly, in both sex groups: 1) drinking was negatively associated with poor SRH, that is, drinkers rated their health as better than did non-drinkers and; 2) middle levels of income were associated with better SRH than were high income levels (>$150,000 Canadian).

Education and vision were significant predictors only for women and, therefore, were excluded from the ‘men’ model. Other variables had very different effects on SRH for each sex: 1) number of chronic conditions, while highly associated for all, was a much stronger predictor of poor SRH in women (PRR = 5.30; 95%CI: 2.44, 11.52 in women; PRR = 3.15; 95%CI: 1.96, 5.07 in men); 2) the detrimental effect of very low income (<$20,000) was strong for men and not significant for women (PRR = 1.75; 95%CI: 1.30, 2.34; PRR = 1.24; 95%CI: 0.96, 1.59; respectively); 3) while for men being underweight was associated with approximately twice the likelihood of reporting poor SRH (PRR = 2.10; 95%CI: 1.20, 3.66), among women, being overweight or obese were stronger predictors; 4) receiving both types of care (formal and informal) was strongly associated with poor SRH for all, but more so for men than women (PRR = 3.27; 95%CI: 2.55, 4.18; PRR = 2.71; 95%CI: 2.20, 3.32; respectively).

In intercept only multilevel models when single clusters were examined and compared wealth explained the greatest proportion of variability in SRH (strongest cluster effect). Almost 21% of differences in SRH was explained via clusters defined by ‘wealth group’ differences, alone. The next strongest cluster effect (5%) was observed for education groups. Cluster effects from sex or rural/urban status groupings were very small, explaining only 0.12 and 0.2%, respectively. To assess intersectionality, after looking at strata defined by combinations of two of the above factors, the largest ICCs were for ‘education and wealth’ and for ‘sex and wealth’ (almost 15%). Adding either of sex or education to wealth grouping factors lowered the cluster effect of wealth from 21% to 15%. Despite very small random effects from ‘rural/urban status’ alone, the largest cluster effect (13.6%) from the strata shaped by three factors was for ‘sex, wealth, and rural/urban status’ and not ‘sex, wealth, and education’, suggesting complex, intersecting impacts from these categories rather than simple additive or multiplicative interaction effects. All cluster effects were verified by calculating MORs (Table 4).

**Table 4:** Cluster effects of sex and social factors and their combinations

Random effect	ICC	*P* value for the random intercept	MOR
Sex	0.1%	0.1408	1.04
Education	4.7%	< 0.0001	1.46
Wealth	20.6%	< 0.0001	2.40
Rural/urban status (RUS)	0.2%	0.1286	1.08
Sex & Education	3.4%	< 0.0001	1.38
Sex & Wealth	14.8%	< 0.0001	2.05
Sex & RUS	0.1%	0.01112	1.06
Education & Wealth	14.9%	< 0.0001	2.06
Education & RUS	3.2%	< 0.0001	1.37
Wealth & RUS	15.3%	< 0.0001	2.08
Sex, Education & Wealth	13.2%	< 0.0001	1.96
Sex, Education & RUS	2.9%	< 0.0001	1.35
Sex, Wealth & RUS	13.6%	< 0.0001	1.98
Education, Wealth & RUS	7.5%	< 0.0001	1.63
Sex, Education, Wealth & RUS	13.4%	< 0.0001	1.97

*ICC* Intra-class Correlation Coefficient, *MOR* Median Odds Ratio

## Discussion

The interplay between who one is (individual characteristics), one’s lived circumstances (social and contextual characteristics), and health is well documented, strong, and of particular importance with aging and concomitant accumulation of opportunities and constraints. The congruence of measures of objective and subjective health is also robust. Less clear is how those interconnections between lived realities, themselves, and individual biology actually shape health. We have gone beyond simply categorizing by sex with its inevitable assumption that, for example, all women are the same and different from all men, and found that intersections of sex and social strata or locations deepen explanations for reported health differences. This is beyond simple interaction analysis that assumes all people within a social location are similar. Guided by social determinants of health theories [24] and conceptual models that explain population variations in SRH [6, 52] we theorized health to be a function of the interplay between sex, individual, social, and contextual factors. Simple regression models that only estimate fixed effects of specified factors are not able to examine this complexity and therefore, an intersectionality approach is warranted and valuable [34, 53].

We started our analyses by constructing well-formulated Poisson regression models. Although diminished by each of chronic conditions, lower social participation, lower wealth, poorer nutrition or hearing, and lower grip strength, overall, the CLSA population of 45+ year old Canadians perceived themselves as healthy.

Some findings, such as greater SRH among those who consume alcohol and middle versus high income groups, were at odds with expectations and existing evidence. While some studies report health benefits associated with lower levels of alcohol consumption [54], from the available data we cannot determine whether there are unmeasured but related characteristics for which these are proxies. For example, individuals with middle income may feel less stress and be more satisfied with their life, and hence perceive their health as better compared to higher income groups. Furthermore, as with any cross-sectional data the possibility of reverse causality cannot be ruled out. We could not, therefore, evaluate whether those with better health consume more alcohol or whether drinking more predicts better self-perceived health.

There were sex differences in self-rated health and circumstances associated with it, with women rating their health more highly than men, overall. Bivariate analysis showed that women were 7% less likely to report poor SRH although this protective effect was only marginally significant. The protective effect of ‘female sex’ increased dramatically to 43% after adjustment for a variety of confounders using the carefully specified model. This suggests that actual (as estimated in the adjusted models) good health perception is much higher in women; however, when the effects of social factors and health behaviours are not taken into account (unadjusted model) most of the effect of sex disappears. In other words, the sex-SRH relationship is strongly influenced by social and behavioral factors. Put another way, it appears to be gender rather than sex that is a strong predictor of SRH. This nuance would not have been apparent if the impact of sex, alone, been considered.

Education, vision, number of chronic conditions, and being overweight were either uniquely or more importantly associated with poor SRH for women than men. Among men, low income, being underweight and needing formal or informal care were the unique or more important characteristics underlying poorer SRH. The differential effect of body weight in men and women may speak to underlying gender stereotypes about weight. For women in the CLSA sample being overweight was perceived as unhealthy. This perception among women, while not surprising, may be incorrect as some studies show that after adjustment for SES factors being overweight is not a risk factor for mortality among Canadians [55]. In men the reverse occurred with underweight status perhaps being interpreted as a marker of frailty and lack of masculinity and, hence, aligning with lower perceptions of health. Education is universally reported as a determinant of health [56], however in our study it was only a significant predictor of SRH among women. We observed that other factors such as wealth and income may have acted as proxies for education in men but not in women suggesting, once again, the gendered nature of health impacts of these social factors.

On their own, sex differences accounted for very little of the observed variability in SRH, while wealth had strong explanatory value. Clusters defined by sex and wealth explained less variability than did wealth, alone, hinting at a relationship between sex and wealth beyond an additive, interactive effect. While belonging to different wealth groups explained a large amount of the variation in SRH, when groupings were further divided to include combinations of sex and wealth that explanatory power decreased. We interpret this as potential evidence of an intersection rather than an interaction between the two categories since this observed effect suggests an interlocking impact of sex and wealth and speaks to the need for and merit of an intersectional approach to analyses. Somehow when the characteristics of sex and wealth defined clusters collectively, the impact on SRH was less than that of wealth, alone, a result that would not have been apparent had analyses considered sex or wealth as independent variables, or interactions between the two. From our research we cannot determine why sex mutes the impact of wealth on health but since we tested for an interaction between these two and that interaction was not statistically significant, we argue that there is a complex interplay between these factors, that is *intersectional* rather than an *interactive*.

We also examined intersections of sex and two additional social locations, education and place of residence. Place, defined as rural/urban status, by itself had only a very small cluster effect (0.02%) but when combined with sex and education the cluster effect increased significantly such that more than 13% of variability in SRH was explained by differences between strata defined by intersections of sex, education, and place. This also suggests that these social factors concurrently impact health in an interconnected way rather than via simple additive or multiplicative interacting effects.

### Strengths and limitations

A key strength of this study is its robust theoretical framework that looks beyond simple risk factor epidemiologic thinking. By utilizing determinants of health and intersectionality theories we have provided evidence that assists in disentangling the complexity underlying self-perceived health in old age. Use of a large, representative and national dataset of 45+ year old Canadians is another strength of the study. Large sample sizes allowed us to examine a number of interactions without diminishing statistical power.

Use of cross-sectional data inherently raises the possibility of reverse causality; we cannot assess whether social adversities preceded poor SRH. The main limitation of the research arises, however, from uncertainty about best methods for evaluating intersectionality. Several methods are suggested for such work [53] however whether any of these is ‘best’ is still debated [57]. We selected MLA because quantification of cluster effects of social strata provides information beyond that obtainable from regular regression analyses and clearly addresses both within and across category (or cluster) variability. At present, there is exploration and debate about best methods to study the relatively novel construct of intersectionality in quantitative health outcomes research.

## Conclusions

After accounting for true confounders, Canadian middle-aged and older women’s perceptions of their health were significantly better than were men’s. In the CLSA population studied, variations in SRH are, however, better explained by considering intersections among sex, wealth, and rural/urban status.

## Supplementary Information

**Additional file 1.**

## Data Availability

The datasets generated and/or analysed during the current study are not publicly available due to Canadian Longitudinal Study on Aging (CLSA) confidentiality policies but are available from the corresponding author on reasonable request.

## Abbreviations

ADL: Activities of daily living
CLSA: Canadian Longitudinal Study on Aging ()
SES: Socio-economic status
SRH: Self-rated health
ICC: Intra-class Correlation Coefficients
MOR: Median Odds Ratio
