# Supplementary material for: The intersectional impact of sex and social factors on subjective health: analysis of the Canadian longitudinal study on aging (CLSA)
Source: BMC Geriatr. 2021 Aug 28;21:473. doi: 10.1186/s12877-021-02412-6 (PMC8399822; doi:10.1186/s12877-021-02412-6)
Supplement: Supplementary file 1 — Additional file 1. [file 12877_2021_2412_MOESM1_ESM.docx]

**Appendix A: Detailed description of some CLSA variables used in this analysis**

**Social support:**

Was measured using the 19-item Medical Outcomes Study (MOS) Social Support Survey^1^ that provides indicators of four subscales of functional social support:

1) Tangible social support - the provision of material aid or behavioral assistance.

2) Affection - involving expressions of love and affection.

3) Positive social interaction - the availability of other persons to positively interact with.

4) Emotional or informational support - the expression of positive affect, empathetic understanding, and the encouragement of expressions of feelings, or the offering of advice, information, guidance or feedback

An overall social support score was obtained by averaging the responses over all 19 items in the questionnaire.

(1) Sherbourne CD, Stewart AL. The MOS social support survey. Soc Sci Med 1991; 32(6):705-714.

**Social participation:**

The measure included eight questions about community-related activities.

In the past 12 months, how often did you participate in…

1) Family or friendship-based activities outside the household?

2) Church or religious activities such as services, committees or choirs

3) Sports or physical activities that you do with other people

4) Educational and cultural activities involving other people such as attending courses, concerts, plays, or visiting museums

5) Service club or fraternal organization activities

6) Neighbourhood, community or professional association activities

7) Volunteer or charity work

8) Any other recreational activities involving other people, including hobbies, gardening, poker, bridge, cards, and other games

**ADL and IADL Questions:**

ADL questions asked about level of help (if any) needed for 1) dressing and undressing, 2) eating, 3) taking care of your own appearance, 4) walking, 5) getting in and out of bed, 6) taking a bath or shower, 7) getting to the bathroom

IADL questions asked about 1) using the telephone, 2) getting to places out of walking distance, 3) shopping for groceries or clothes, 4) preparing own meals, 5) doing housework, 6) taking own medicine, 7) handling own money

**Depression**:

Was assessed using the Center for Epidemiological Studies Short Depression Scale (CES-D-10) questions with options of ‘All of the time (5-7days)’, ‘Occasionally (3-4 days)’,’Some of the time (1-2 days)’, ‘Rarely or never (less than 1 day)’, ‘Don’t know, no answer’, and ‘Refused’. Participants were to select the answer that most applies to how they have felt over the past week:

1) How often were you bothered by things that usually don’t bother you?

2) How often did you have trouble keeping your mind on what you were doing?

3) How often did you feel depressed?

4) How often did you feel that everything you did was an effort?

5) How often did you feel hopeful about the future?

6) How often did you feel fearful or tearful?

7) How often was your sleep restless?

8) How often were you happy?

9) How often did you feel lonely?

10) How often did you feel that you could not “get going”?
